# Preclinical Evaluation of Engineered Oncolytic Herpes Simplex Virus for the Treatment of Pediatric Solid Tumors

**DOI:** 10.1371/journal.pone.0086843

**Published:** 2014-01-30

**Authors:** Michael L. Megison, Lauren A. Gillory, Jerry E. Stewart, Hugh C. Nabers, Elizabeth Mroczek-Musulman, Alicia M. Waters, Jennifer M. Coleman, Virginia Kelly, James M. Markert, G. Yancey Gillespie, Gregory K. Friedman, Elizabeth A. Beierle

**Affiliations:** 1 Department of Surgery, Division of Pediatric Surgery, University of Alabama, Birmingham, Birmingham, Alabama, United States of America; 2 Department of Pathology, Children’s of Alabama, Birmingham, Alabama, United States of America; 3 Department of Surgery, Division of Neurosurgery, University of Alabama, Birmingham, Birmingham, Alabama, United States of America; 4 Department of Pediatrics, Division of Hematology/Oncology, University of Alabama, Birmingham, Birmingham, Alabama, United States of America; University of Pittsburgh School of Medicine, United States of America

## Abstract

Recently, investigators showed that mice with syngeneic murine gliomas that were treated with a neuroattenuated oncolytic herpes simplex virus-1 (oHSV), M002, had a significant increase in survival. M002 has deletions in both copies of the γ_1_34.5 gene, enabling replication in tumor cells but precluding infection of normal cells. Previous studies have shown antitumor effects of other oHSV against a number of adult tumors including hepatocellular carcinoma and renal cell carcinoma. The purpose of the current study was to investigate the oncolytic potential of M002 against difficult to treat pediatric liver and kidney tumors. We showed that the oHSV, M002, infected, replicated, and decreased cell survival in hepatoblastoma, malignant rhabdoid kidney tumor, and renal sarcoma cell lines. In addition, we showed that in murine xenografts, treatment with M002 significantly increased survival and decreased tumor growth. Finally, these studies showed that the primary entry protein for oHSV, CD111 (nectin-1) was present in human hepatoblastoma and malignant rhabdoid kidney tumor specimens. We concluded that M002 effectively targeted these rare aggressive tumor types and that M002 may have potential for use in children with unresponsive or relapsed pediatric solid tumors.

## Introduction

Despite major advances over the past 20 years in the treatment of pediatric malignancies, there remain a number of pediatric solid tumors that have limited therapies in the face of unresponsive or relapsed disease. Many of these tumors involve solid organs such as the liver or kidneys, and include hepatoblastoma, malignant rhabdoid renal tumors, and non-osseous sarcomas. Successful management of these malignancies will require innovative and novel therapies.

Hepatoblastoma, the most common pediatric hepatic malignancy, accounts for about 50% of malignant liver tumors in children [1]. More than half of the children presenting with hepatoblastoma have advanced or metastatic disease at the time of diagnosis [2], and survival remains poor for those with unresectable or recurrent disease [3]. Malignant rhabdoid kidney tumors (MRKT) are another type of aggressive pediatric solid tumor. These rare renal malignancies comprise 2% of pediatric kidney tumors [4]. Most MRKTs are diagnosed in children less than 4 years of age and more than half of the children present with distant metastasis [5], [6]. The overall ten year survival for MRKTs is less than 30% despite the use of multimodality therapy including surgical resection, chemotherapy and radiotherapy [5], [7], and is even more dismal in neonates, where the survival is reported to be less than 10% [7]. Finally, solid organ sarcomas are some of the most rare and difficult solid tumors to treat in children. These include extra-osseous Ewing’s sarcomas and primitive neuroectodermal tumors (PNET), which are both highly aggressive and carry poor prognoses [8]. Up to 50% of patients presenting with solid organ sarcomas have metastases at diagnosis, and the 5-year disease free survival rate is less than 50% [9]–[11].

The *in vitro* and *in vivo* use of oncolytic viruses have been described for hepatocellular carcinoma [12], [13] and renal cell carcinoma [14]. Kuroda et al demonstrated the increased replication of a transcriptionally targeted oncolytic herpes simplex virus vector, bM24-TE, in several tumor types with highly activated β-catenin/Tcf signaling, including HepG2 hepatocellular carcinoma cells that resulted in a significant decrease in HepG2 cell survival [12]. Argnani and colleagues infected Hep3B, HepG2 and HuH-7 hepatocellular carcinoma cell lines with a recombinant HSV and found a significant decrease in cell survival at low multiplicity of infection (MOI) [13]. In addition, they showed a decrease in tumor volume in HuH-7 hepatocellular xenografts [13]. Fu et al showed two oncolytic herpes simplex viruses, Synco-2D and FusOn-H2, inhibited renal cell carcinoma cell survival *in vitro*. In addition, they also found that intra-tumoral or intravenous injection of these viruses decreased tumor xenograft growth and increased animal survival [14].

In our laboratory, we are currently conducting investigations with the genetically-engineered oncolytic herpes simplex virus, M002, which has been previously described [15], [16]. HSV-1 (F) strain is a low passage clinical isolate used as the prototype HSV-1 strain in our series. Virus R3659, the parent virus for M002, has been described [17]. Briefly, R3659 was the parent virus for M002 with the thymidine kinase gene inserted into deleted regions of both γ_1_34.5 loci and a deletion in the native thymidine kinase locus. M002 is a conditionally replication-competent mutant herpes simplex virus expressing both subunits of murine IL-12 (mIL-12) under the transcriptional control of the murine Early-growth response-1 promoter (Egr-1); two copies of the entire construct are present, with a single copy inserted into each of the γ_1_34.5 loci; the native thymidine kinase gene is restored. Collaborators at our institution have described the use of M002 for the successful treatment of murine gliomas [15], [16]. Survival of syngeneic animals following intracranial injection of Neuro2a murine tumor cells was significantly longer after intratumoral injection with M002, compared to that of animals treated with vehicle [16]. Also, we have recently noted that M002 was effective in decreasing cell survival and xenograft tumor growth in another pediatric solid tumor, neuroblastoma [18]. These previous studies led us to hypothesize that M002 would be cytotoxic for pediatric solid tumors of the liver and kidney.

## Materials and Methods

### Cells and Cell Culture

All cell lines were cultured under standard conditions at 37°C and 5% CO_2_. The human hepatoblastoma cell line, HuH6, was a generous gift from Dr. Thomas Pietschmann (Hannover, Germany), and has been previously described [19]. The HuH6 cell line was maintained in Dulbecco’s Modified Eagle’s Medium with 10% fetal bovine serum (FBS) (Hyclone, Suwanee, GA), 1 µg/mL penicillin (Gibco, Carlsbad, CA) 1 µg/mL streptomycin (Gibco), and 2 mM L-glutamine (Thermo Fisher Scientific, Rockford, IL). The malignant renal rhabdoid tumor cell line, G401 (CRL-1441, ATCC) [20], was maintained in McCoy’s medium containing 10% non-heat-inactivated FBS (Hyclone), 1 µg/mL penicillin/streptomycin (Gibco), and 2 mM L-glutamine (Fisher). The renal Ewing sarcoma cell line, SK-NEP-1 (HTB-48, American Type Culture Collection, ATCC, Manassas, VA) [21], was maintained in McCoy’s medium (30-2007, ATCC) containing 15% FBS (Hyclone), 1 µg/mL penicillin/streptomycin (Gibco) and 2 mM L-glutamine (Thermo Fisher Scientific). Due to their non-adherent nature, SK-NEP-1 cells were passed as needed by centrifugation at 300 rpm for 5 minutes, aspiration of the supernate to remove cellular debris, and resuspension of the pelleted live cells in flasks containing growth medium. Vero cells were obtained from ATCC (CCL-81) and maintained in modified Eagle’s medium (Corning CellGro, Manassas, VA) containing 7% FBS (Hyclone), 1 µg/mL penicillin/streptomycin (Gibco) and 2 mM L-glutamine (Thermo Fisher Scientific).

### Antibodies

Polyclonal rabbit anti-PVRL-1 (CD111) and anti-HSV-1 were from Abcam (ab71512, Abcam, Cambridge, MA) and Biogenex (PU084-UP, Biogenex, Fremont, CA), respectively. Mouse monoclonal anti-GAPDH was from Millipore (MAB374, EMD Millipore, Billerica, MA).

### Human Tissue Specimens

Formalin-fixed, paraffin-embedded hepatoblastoma and malignant rhabdoid kidney tumor specimens were obtained after approval by the University of Alabama, Birmingham, Institutional Review Board for Human Use (X100930009 and X110825022, respectively) and waiver of informed consent.

### Virus

Stock M002 virus was titered to confirm viral concentration prior to use in experiments. To titer the virus, Vero cells were plated in 24-well culture plates at 1.5×10^5^ cells per well and allowed to attach and form a confluent monolayer. Ten-fold dilutions of stock virus in infection medium (MEM) were applied to the Vero cells for 2 hours, the innoculum removed, and the plates washed with media. Following another 48 hours of incubation, May-Grunwald Stain, 1.25 g (205435, Sigma-Aldrich Corp., St. Louis, MO) in 500 mL methanol, was applied for 10 minutes; the plates washed and allowed to dry overnight. Plaques were counted and the titer was calculated and reported as plaque forming units (PFU)/mL.

### Immunohistochemistry

Formalin-fixed, paraffin-embedded human tumor or xenograft tumor specimens were cut into 6 µM sections, and baked at 70°C for 1 hour, deparaffinized, rehydrated and steamed. The sections were then quenched with 3% hydrogen peroxide and blocked with blocking buffer (BSA, powdered milk, Triton X-100, PBS) for 30 minutes at 4°C. The primary rabbit polyclonal antibodies, anti-CD111 (1∶200, Abcam) or anti-HSV-1 (1∶250, Biogenex) were added and incubated overnight at 4°C. After washing with PBS, the anti-rabbit HRP secondary antibody (Jackson ImmunoResearch Laboratories, Inc., West Grove, PA) was added 1∶250 dilution for 1 hour at 22°C. For Ki67 staining, the primary anti-Ki67 rabbit polyclonal antibody (ab15580, Abcam) was added 1∶200 dilution and incubated overnight at 4°C. After washing with PBS, the donkey anti-rabbit secondary antibody was added 1∶400 dilution (Jackson ImmunoResearch Laboratories) for 1 hour at 22°C. The staining reactions were developed with VECTASTAIN Elite ABC kit (PK-6100, Vector Laboratories, Burlingame, CA), TSA™ (biotin tyramide reagent, 1∶400, PerkinElmer, Inc., Waltham, MA) and DAB (Metal Enhanced DAB Substrate, Thermo Fisher Scientific). Slides were counterstained with hematoxylin. Negative controls [rabbit IgG (1 µg/mL, EMD Millipore)] were included with each run. For hematoxylin and eosin staining, slides were cut and baked as described above and standard H&E staining methods were utilized.

### Immunohistochemical Scoring

A single board-certified pathologist (E.M.M.) blinded to the patients (human tissue) or treatment groups (murine tumors) reviewed and scored immunohistochemistry for CD111 based upon the intensity of staining and the percentage of tumor cells within each category. Intensity staining was graded from 0 to 3 (0, none; 1, weak; 2, moderate; 3, strong), and multiplied by the percentage of cells with that staining. For example, if the specimen showed moderate staining (2) in 40% of the cells, the stain score would be 80 (2×40 = 80).

Quantification of Ki67 staining (proliferative activity) was completed by a single board-certified pathologist (E.M.M.) blinded to the samples. The area chosen for analysis was always the area of greatest immunoreactivity in the specimens. Five hundred cells were counted from this area and the ratio of immunopositive cells to total cells was reported as percent positive staining. All immunopositive cells were counted without regard to the intensity of the stain [22].

### Immunoblotting

Western blots were performed as previously described [23]. Briefly, whole cell lysates or homogenized xenograft specimens were isolated using RIPA lysis buffer [10 mM Tris base pH 7.2, 150 mM NaCl, 1% Na-deoxycholate, 1% Triton X-100, 0.1% sodium dodecyl sulfate (SDS)] supplemented with protease inhibitors (Sigma), phosphatase inhibitors (Sigma) and phenylmethanesulfonylfluoride (10 µg/mL). Lysates were cleared by centrifugation at 14 000 rpm for 30 min at 4°C. Protein concentrations were determined using BCA Protein Assay Reagent (Pierce, Rockford, IL) and proteins separated by electrophoresis on sodium dodecyl sulfate polyacrylamide (SDS-PAGE) gels. Antibodies were used according to manufacturers’ recommended conditions. Molecular weight markers (Precision Plus Protein Kaleidoscope Standards, Bio-Rad, Hercules, CA) were used to confirm the expected size of the target proteins. Immunoblots were developed with Luminata Classico or Crescendo ECL (EMD Millipore). Blots were stripped with stripping solution (Bio-Rad) at 37°C for 15 minutes and then reprobed with selected antibodies. Equal protein loading was confirmed with immunoblotting with antibody to GAPDH.

### Flow Cytometry Analysis (FACS)

Cellular expression of CD111 and CD112 was measured with flow cytometry. HuH6 and G401 cells were harvested with trypsin and SK-NEP-1 cells by agitation and then centrifuged at 900 rpm for 4 minutes. Previous work in our laboratory demonstrated no effect of trypsin upon CD111 expression as detected by FACS (Figure **S1**). The cell pellet was pipetted with autoMACS® running buffer (130-091-221, Miltenyi Biotec, Bergisch Gladbach, Germany) to obtain a single cell suspension. Cells were blocked with 20 µL FcR Blocker (120-000-442, Miltenyi Biotec) and cells with blocker alone served as negative controls. Phycoerythrin (PE) conjugated anti-human CD111 or CD112 antibodies (340403, 337410, respectively, Biolegend, San Diego, CA) were added and cells incubated at 4°C in the dark for 20 minutes. Cells were again centrifuged and a single cell suspension was obtained with autoMACS® buffer (Miltenyi Biotec). Cells were analyzed with fluorescence-activated cell sorting (FACS) using a BD LSR II Flow Cytometer (BD Biosciences, San Jose, CA). Data were analyzed with FlowJo v10.0.6 (Tree Star Inc., Ashland, OR).

### Viral Replication

M002 viral replication was confirmed *in vitro* using infectivity assays. Single step viral assays were performed as previously described [24]. Briefly, cells were plated and allowed to attach overnight and were then infected with M002 at a multiplicity of infection (MOI) of 10 PFU/cell for 2 hours. After 12 and 24 hours, the cells were harvested by adding equal volumes of sterile milk and freezing at −80°C. Plates were thawed at 37°C and underwent two more cycles of freeze/thaw. Cells and supernates were collected, milk stocks sonicated for 30 seconds, and the titers of progeny virions were determined on monolayers of Vero cells. The average number of PFU/mL was calculated from quadruplicate wells. For multi-step viral recovery experiments, cells were grown to confluence and then infected with M002 at a MOI of 0.1 PFU/cell. The media was collected at 6, 24, 48, and 72 hours post-infection. For each timepoint, the titers of progeny virions in the supernate were determined on monolayers of Vero cells, and the average number of PFU/mL was calculated from quadruplicate wells.

### ELISA

Production of murine IL-12 by the recombinant virus was quantified using a murine specific IL-12 ELISA kit (EMIL12TOT, Thermo Fisher Scientific). Ninety-six well plates were seeded with 1.5×10^4^ cells per well for 24 hours and then treated with media alone or M002. After 48 hours of incubation, the supernates were collected and analyzed with ELISA according to the manufacturer’s protocol.

### Cell Viability Assays

Equal numbers of cells were plated, treated with increasing MOI of M002, and cell viability was measured with alamarBlue® assays [25]. In brief, 1.5×10^4^ cells per well were plated in 96-well culture plates and after 24 hours were treated with 100 µL of saline or a graded series of dilutions of M002 for 72 hours. Following treatment, 10 µL of alamarBlue® dye (Invitrogen, Life Technologies, Grand Island, NY) was added to each well. After 4–6 hours, the absorbance at 595 nm was measured using a kinetic microplate reader (BioTek Gen5, BioTek Instruments, Winooski, VT). Virus cytotoxicity at each dilution was measured by the reduction in the color change compared with that seen in the saline treatment group (100%) viability. These values were plotted to yield an estimate of the numbers of PFUs of M002 needed to kill 50% of the cells by 72 hours (PFU/LD_50_).

### Ethics Statement

All animal experiments were performed after obtaining protocol approval by the University of Alabama, Birmingham Animal Care and Use Committee (130409363), and in compliance with the recommendations in the Guide for the Care and Use of Laboratory Animals of the National Institutes of Health. The human subject samples were obtained after protocol approval by the University of Alabama, Birmingham Institutional Review Board for Human Use (X100930009 and X110825022) under waiver of informed consent.

### *In vivo* Tumor Growth

Six week old, female, athymic nude mice were utilized (Harlan Laboratories, Inc., Chicago, IL). The mice were maintained in the SPF animal facility with standard 12 hour light/dark cycles and allowed chow and water *ad libitum*. All experiments were performed after obtaining protocol approval by the University of Alabama, Birmingham Institutional Animal Care and Use Committee (130409363) and in compliance with the institutional, national and NIH animal use guidelines. At the completion of the experiments, euthanasia was accomplished according to American Association for Laboratory Animal Science (AALAS) guidelines utilizing compressed CO_2_ gas in cylinders in the home cage followed by bilateral thoracotomy. For the first set of experiments, a flank xenograft model was utilized. Human hepatoblastoma cells, HuH6 [2.5×10^6^ cells in Matrigel™ (BD Biosciences)] were injected into the subcutaneous space of the right flank. Tumors were measured twice weekly with a caliper and volume in mm^3^ was calculated using the standard formula [(width)^2^×length]/2, where width was the smaller diameter. Once flank tumors reached a volume of 250 mm^3^, the tumors were injected with either control vehicle (PBS +10% glycerol/50 µL, n = 10) or M002 oncolytic herpes simplex virus (1×10^7^ PFU/50 µL, n = 10). This virus concentration was based upon previous investigations with this virus [16], [18]. Half of the control tumors and half of the M002 treated tumors received a low dose of external beam irradiation (3 Gy) within 24 hours of M002 treatment. When flank tumors reached the parameters allowed by the IACUC protocol, the animals were euthanized and the tumors harvested. Survivors were followed over time to watch for tumor growth.

In the next group of xenograft experiments, human malignant renal rhabdoid tumor cells, G401 [2.5×10^6^ cells in Matrigel™ (BD Biosciences)] were injected into the subcutaneous space of the right flank. Tumors were measured twice weekly with calipers and once they reached a volume of 250 mm^3^, the tumors were injected with either control vehicle (PBS +10% glycerol/50 µL, n = 5) or M002 oncolytic herpes simplex virus (1×10^7^ PFU/50 µL, n = 10). The control tumors and half of the M002 treated tumors received a low dose of external beam irradiation (3 Gy) within 24 hours of M002 treatment. Tumors were measured twice weekly with a caliper and volume in mm^3^ was calculated as above. When flank tumors reached the parameters allowed by IACUC protocol, the animals were euthanized and the tumors harvested. Survivors were followed for over 3 months to watch for tumor growth.

For the final set of *in vivo* experiments, human renal Ewing sarcoma cells, SK-NEP-1 (1.5×10^6^ cells) were injected into the subcapsular space of the left kidney. After three weeks, the renal tumors were injected with either control vehicle (PBS +10% glycerol/50 µL, n = 6) or M002 oncolytic herpes simplex virus (1×10^7^ PFU/50 µL, n = 7). After two weeks of treatment, the animals were euthanized and the kidney tumors were harvested, weighed, and measured with a caliper. Tumor volume in mm^3^ was calculated using the standard formula [(width)^2^×length]/2, where width was the smaller diameter. Control nude mouse kidney weights and volumes were obtained from animals that did not undergo injection of tumor cells.

### Data Analysis

Experiments were repeated at least in triplicate, and data reported as mean ± standard error of the mean (SEM). Student’s t-test or ANOVA was used as appropriate to compare data between groups and log-rank test was used to determine survival significance. Statistical analyses were completed using SigmaPlot™ 12 software (SyStat Software, Inc., San Jose, CA). Statistical significance was determined at the p≤0.05 level.

## Results

### CD111 Expression

**Figure 1:**
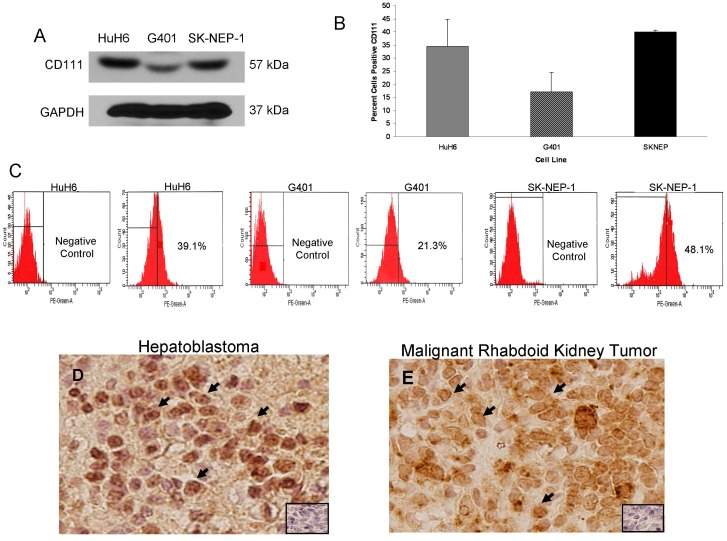
CD111 expression in hepatoblastoma and rare pediatric renal tumor cell lines and specimens. **A** Immunoblotting with CD111 specific antibody demonstrated that CD111 was present in whole cell lysates of HuH6, G401, and SK-NEP-1 cell lines. Detection of GAPDH was used to confirm equal protein loading. **B** HuH6, G401, and SK-NEP-1 cells were stained with fluorescence antibody for CD111. CD111 staining in these cell lines was detected in amounts that were consistent with those seen by immunoblotting (**A**). Stained cells were counted with flow cytometry and representative scatterplots presented for both negative controls and stained cells (**C**). **D** Immunohistochemistry staining with CD111 antibody was performed on 9 formalin-fixed, paraffin-embedded human hepatoblastoma and 12 malignant rhabdoid kidney tumor specimens. Representative photomicrographs presented at 40×, and negative controls were included with each (*small box inserts*). There was no CD111 staining detected in the negative controls. **D** Cellular staining for CD111 (*black arrows*) was present in over half of the hepatoblastoma specimens examined and was not limited to background stromal staining. **E** Staining for cellular CD111 (*black arrows*) was detected in the majority of MRKT specimens.

CD111 [poliovirus receptor-related protein 1 (PRR1, nectin-1)] is a cell surface receptor that is the primary entry mediator used by HSV-1 for cellular entry [26]. Immunoblotting with CD111 specific antibody demonstrated that CD111 was present in whole cell lysates of HuH6, G401, and SK-NEP-1 cell lines (Figure 1. **A**). Further, CD111 staining in these cell lines was detected by flow cytometry in amounts that were consistent with those seen by immunoblotting (Figure 1. **B**). Representative FACS scatterplots for these cell lines presented in Figure 1. **C**. To provide the rationale for clinical applications of oHSV therapy for these aggressive pediatric solid tumors, we performed CD111 immunostaining on human hepatoblastoma and malignant rhabdoid kidney tumor (MRKT) specimens. The CD111 staining was scored by a pathologist (E.M.M.) blinded to the specimens and the mean stain scores were compiled based upon stain intensity. CD111 staining was positive in over half of the hepatoblastoma specimens studied, and in 92% of the MRKT specimens (Table 1). Representative photomicrographs were presented at 40× (Figure 1. **D**, **E**). There was cellular CD111 staining in both tumor types (Figure 1. **D**, **E**, *black arrows*) and negligible background staining noted in the negative control (rabbit IgG) (Figure 1. **D**, **E**, *small box inserts*). These data clearly demonstrated that the cell lines of interest and a number of human tumor specimens examined expressed the HSV entry receptor, CD111.

**Table 1:** Immunohistochemical staining CD111^+^ human tumors.

Tumor Type	N	CD111 PositiveStain (%)	Median StainScore (Range)
Hepatoblastoma	9	5 (56%)	10 (0–140)
MRKT	12	11 (92%)	102.5 (0–300)

### *In vitro* Viral Infectivity and Cell Sensitivity to M002

**Figure 2:**
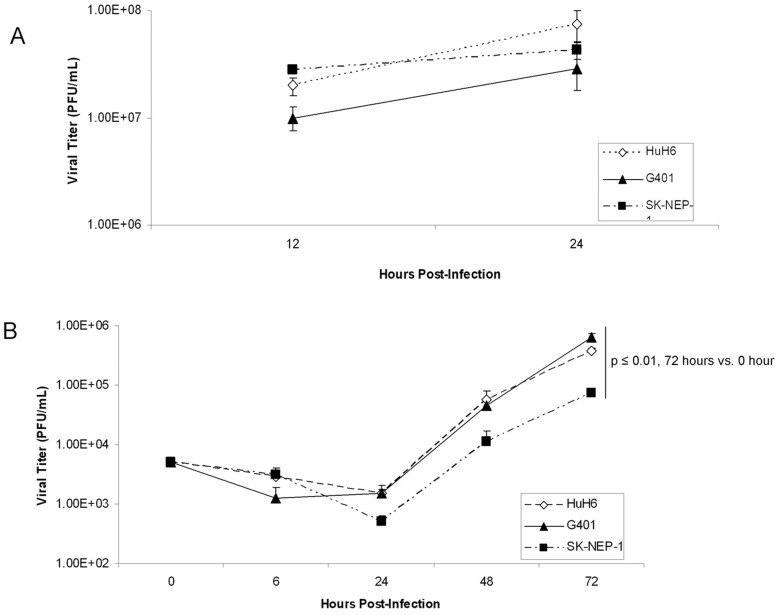
M002 infection. **A** Single-step *in vitro* replication of M002. Monolayers of HuH6, G401 or SK-NEP-1 cells were infected with M002 at a MOI of 10 PFU/cell. Replicate cultures were harvested at 12 and 24 hours post-infection and virus titers determined on Vero cell monolayers. Mean virion yields were determined in four replicates at each time point and standard error of the mean was determined. **B** Multi-step replication of M002. Monolayers of HuH6, G401 or SK-NEP-1 cells were infected with M002 at a MOI of 0.1 PFU/cell, and at 6, 24, 48, 72 hours post-infection, supernates were collected and virus titers determined on Vero cell monolayers. Mean virion yields were determined in four replicates at each time point and standard error of the mean was determined. M002 replicated nearly 2 logs higher than control at 72 hours post-infection.

The *in vitro* replication rates of M002 were evaluated in the cell lines using single and multi-step viral recovery experiments. For the single step experiments, the HuH6, G401, and SK-NEP-1 cell lines were infected with M002 at a MOI of 10 PFU/cell. By 12 hours post-infection, there were significant viral titers noted in all three cell lines (Figure 2. **A**) that continued to increase at 24 hours post-infection (Figure 2. **A**). For multi-step viral recovery, monolayers of the three cell lines were infected with M002 at a MOI of 0.1 PFU/cell, and at 6, 24, 48 and 72 hours post-infection, viral replication was determined. As shown in Figure 2. **B**, after 72 hours of infection, M002 replicated to a titer significantly higher in all three cell lines compared to that of time zero.

**Figure 3:**
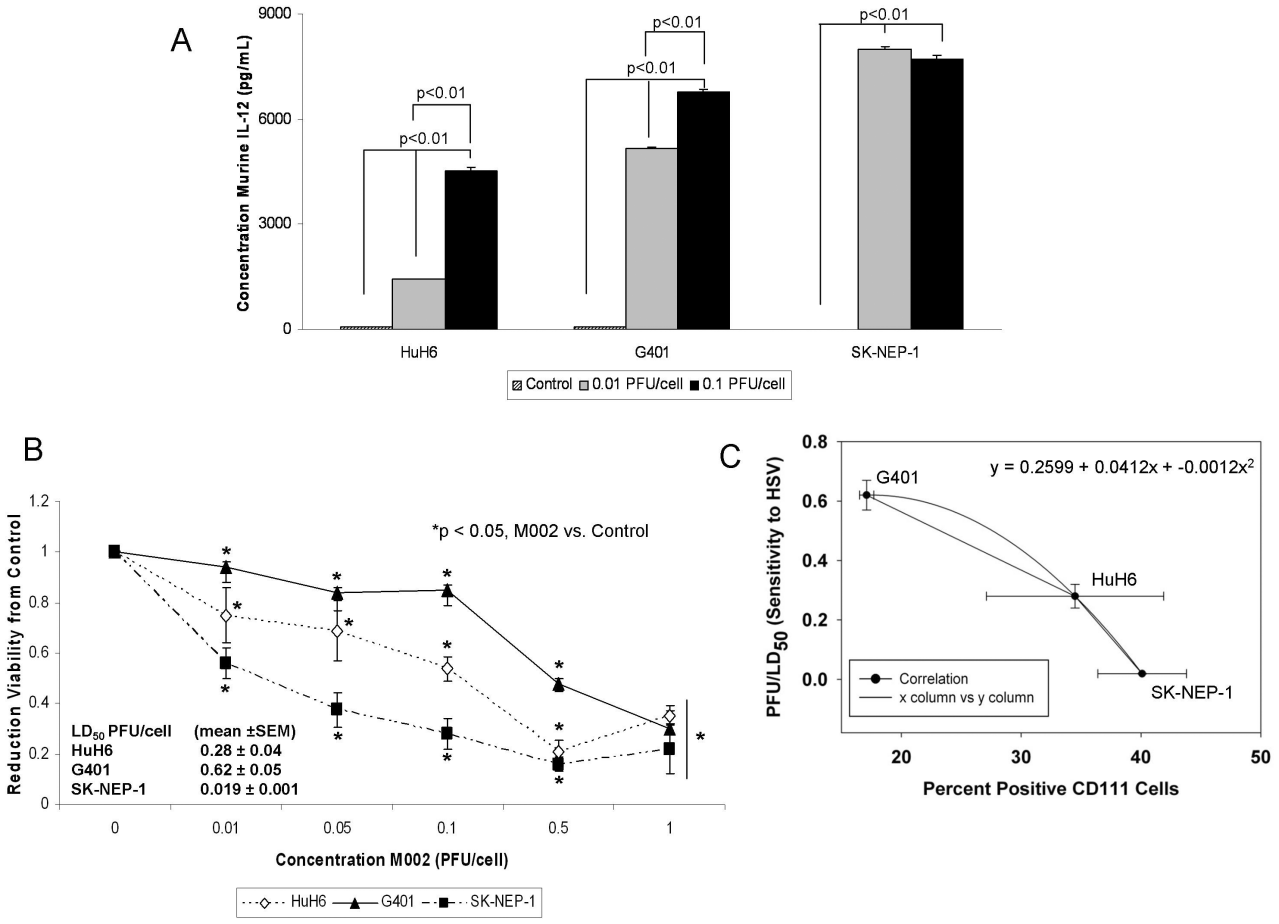
M002 infection and cell survival. **A** To further verify infectivity, murine IL-12 production was determined in HuH6, G401 and SK-NEP-1 cell lines following treatment with M002 oHSV. Cell lines were infected with M002 at 0, 0.01 or 0.1 PFU/cell. After 48 hours, the supernates were collected and concentrations of murine IL-12 determined by ELISA. Data reported as mean ± standard error of the mean. There was a significant increase in murine IL-12 production in all cell lines even at the lower MOI of 0.01 PFU/cell. **B** HuH6, G401 and SK-NEP-1 cell lines were treated with M002 at increasing MOI. After 72 hr of treatment, cell viability was measured with alamarBlue™ assays. Data reported as mean ± standard error of the mean. There was a significant decrease in viability in all cell lines following M002 treatment. The LD_50_ was calculated for each cell line for M002 (PFU/cell). **C** A comparison was made between percent cells staining for CD111 and the sensitivity of the cell lines to M002. There was a non-linear, inverse correlation between the two variables. The straight lines represent the slope between two points and the curved line the correlation calculated with the three points, represented by the polynomial equation. The bars represent the SEM for the data points in both the x and y axis.

Since M002 was genetically engineered to produce murine IL-12, to further verify viral infection, we sought to determine the extent to which the infected human cell lines would produce the encoded foreign murine IL-12 protein. The HuH6, G401 and SK-NEP-1 cells were infected with M002 at 0.01 or 0.1 PFU/cell. After 48 hours of infection, the supernates were collected and a commercially available murine IL-12 ELISA kit was utilized to detect IL-12 production. M002 infection of all cell lines resulted in a significant increase in the production of murine IL-12, even at the lower MOI of 0.01 PFU/cell (Figure 3. **A**). In the HuH6 cell line, infection with 0.01 PFU/cell for 48 hours resulted in a concentration of murine IL-12 of 1422±15 pg/mL, significantly greater than baseline concentration of 60±0.5 pg/mL (p≤0.01) (Figure 3. **A**). Similar findings were seen with the G401 cell line; infection with 0.01 PFU/cell for 48 hours resulted in a concentration of murine IL-12 of 5152±37 pg/mL significantly greater than baseline concentration of 61±5 pg/mL (p≤0.01) (Figure 3. **A**). These trends continued for the SK-NEP-1 cell line also (Figure 3. **A**). In addition, increasing the MOI of the M002 by a factor of 10 resulted in a significant increase in the production of murine IL-12 in the HuH6 and the G401 cell lines compared to the lower MOI (Figure 3. **A**). These same results were not noted with the SK-NEP-1 cell line (Figure 3. **A**), likely since the IL-12 cytokine produced had reached the upper limits of detection of the ELISA kit at the lower MOI of 0.01 PFU/cell. These ELISA studies were utilized only to verify virus infection.

We then investigated cell viability following M002 treatment. The HuH6, G401 and SK-NEP-1 cell lines were treated with M002 at increasing MOI (PFU/cell) and cell viability was measured with alamarBlue® assays after 72 hours of treatment. Viability was decreased in all three cell lines following M002 treatment, and this decrease was statistically significant beginning at the lowest MOI of 0.01 PFU/cell of M002 (Figure 3. **B**). The lethal dose of virus that resulted in 50% cell death (PFU/LD_50_) for each of the cell lines was calculated (Figure 3. **B**). Since CD111 is the primary entry mediator used by HSV-1 [26], a comparison was made between the percent positive CD111 cells and the sensitivity of the cell lines to M002. There was a non-linear, inverse relation between the PFU/LD_50_ and the amount of CD111 staining present in the cell lines (Figure 3. **C**).

### *In vivo* Tumor Studies

**Figure 4:**
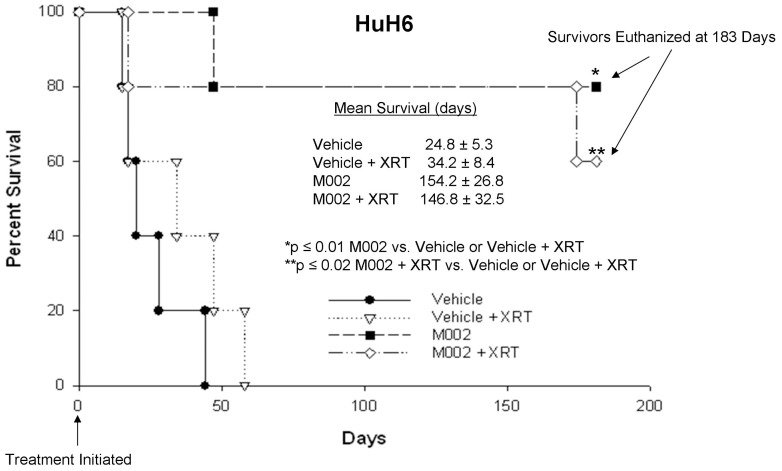
M002 treatment of hepatoblastoma xenografts. HuH6 human hepatoblastoma tumor cells (2.5×10^6^ cells) in Matrigel™ were injected into the right flank of female athymic nude mice. Once tumors reached 250 mm^3^, animals received an intra-tumoral injection of vehicle [PBS +10% glycerol, 50 µL (n = 10)] or M002 virus [1×10^7^ PFU/50 µL (n = 10)]. Following injection, half of the animals in each group were treated with 3 Gy irradiation to the tumor. Kaplan-Meier graph for animal survival. Animals treated with M002 alone or with M002+ XRT had a significant increase in survival over those treated with vehicle alone or with vehicle+XRT. There was no significant survival advantage seen with the addition of XRT to either the vehicle treated animals or the M002 treated animals.

**Figure 5:**
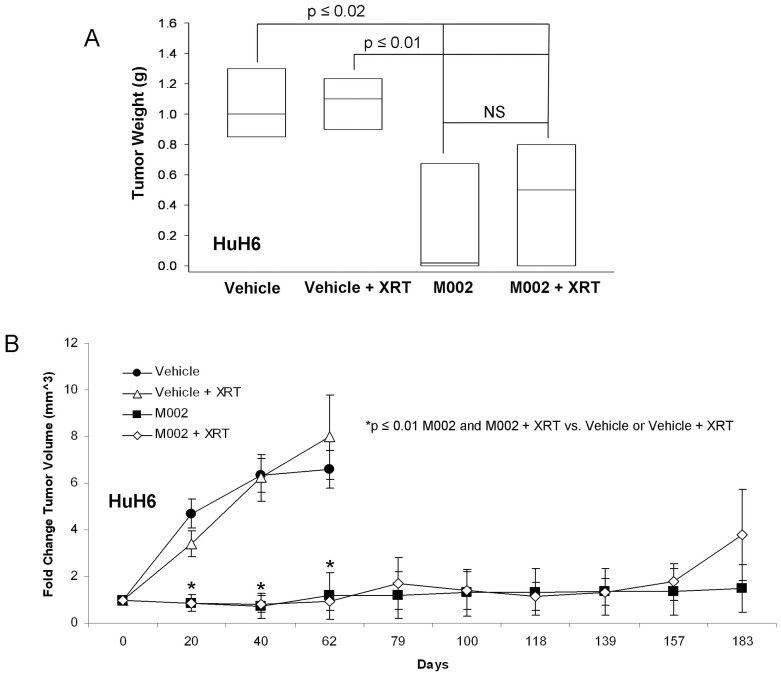
**A** The HuH6 tumor xenografts (as described in Figure 4) were weighed at euthanasia. The mean tumor weights from animals treated with M002 alone or M002+ XRT were significantly smaller than weights of the tumors from animals treated with vehicle or vehicle+XRT. Bars represent mean. Tumor weights were not altered by the addition of XRT to either the vehicle or M002 treatments. **B** HuH6 tumor xenograft volumes were measured biweekly with calipers [(width)^2^×length]/2. Data reported as mean fold change in tumor volume ± standard error. In the HuH6 xenografts, there was a significant decrease in tumor growth that began at day 7 following treatment that continued to the end of the study. The addition of low dose XRT to either the vehicle or M002 treatments had no significant effect upon tumor growth.

It must be stressed that there are no syngeneic animal models available for these tumor types. Therefore, to determine the *in vivo* effects of M002 treatment on hepatoblastoma, a nude mouse xenograft model of HuH6 was utilized. HuH6 cells [2.5×10^6^ cells in Matrigel™ (BD Biosciences)] were injected into the subcutaneous space of the right flank. Once xenografts reached 250 mm^3^, tumors were injected with vehicle (PBS+glycerol, 50 µL, n = 10) or M002 (1×10^7^ PFU/50 µL, n = 10). Since the addition of low dose radiation has been shown to increase the activity of oHSV in malignant gliomas [27], [28], [29], we also examined whether the addition of low dose radiation would enhance the efficacy of M002 in the hepatoblastoma xenografts. Therefore, half of the animals in each group also received a low dose of irradiation to the tumor immediately following injection with vehicle or M002. Three gray (3 Gy, XRT) was chosen as the dose since other investigations have shown that HSV replication increased in a dose dependent fashion following irradiation with 2 to 5 Gy, with no additional effects seen after 5 Gy [29]. Tumor volumes were measured biweekly with calipers. Animals were euthanized when tumor parameters reached those allowed by IACUC protocol, or at 183 days when the experiment was terminated. Animals treated with M002 alone or with M002+ XRT had a significant increase in survival over those treated with vehicle alone or with vehicle+XRT (Figure 4.). There was no significant survival advantage seen with the addition of XRT to either the vehicle treated animals or the M002 treated animals (Figure 4.). The HuH6 tumor xenografts were weighed at euthanasia and those treated with M002 alone or M002+ XRT were significantly smaller than tumors from animals treated with vehicle or vehicle+XRT (Figure 5. **A**). We also examined fold change in tumor volume which was defined as the tumor volume at the specified time divided by the initial tumor volume. In the HuH6 xenografts, there was a significant decrease in tumor growth following M002 treatment either with or without XRT, which continued to the end of the study (Figure 5. **B**). Again, the addition of low dose XRT to either the vehicle or M002 treatments had no significant effect upon tumor growth (Figure 5. **B**).

**Figure 6:**
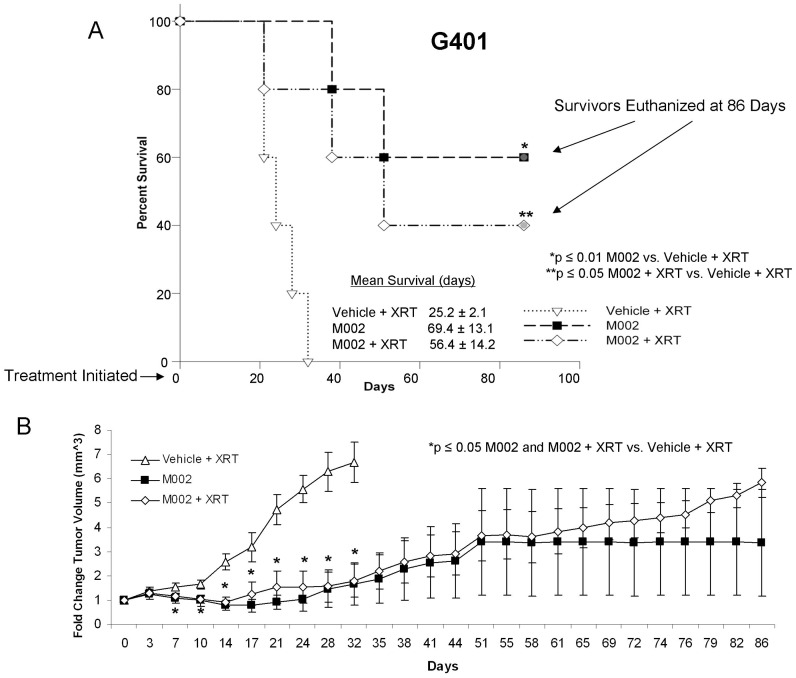
M002 treatment of malignant rhabdoid kidney tumor xenografts. G401 human MRKT cells (2.5×10^6^ cells) in Matrigel™ were injected into the right flank of female athymic nude mice. Once tumors reached 250 mm^3^, animals received an intra-tumoral injection of vehicle [PBS +10% glycerol, 50 µL (n = 5)] or M002 virus [1×10^7^ PFU/50 µL (n = 10)]. Following injection, the vehicle treated and half of the M002 treated animals were given 3Gy irradiation to the tumor. **A** Kaplan-Meier graph for animal survival. Animals treated with M002 alone or with M002+ XRT had a significant increase in survival over those treated with vehicle+XRT. There was no significant survival advantage seen with the addition of XRT to the M002 treatment. **B** Tumor volumes were measured twice weekly [(width)^2^×length]/2. Data reported as mean fold change in tumor volume ± standard error. In the M002 treated G401 xenografts, there was a significant decrease in tumor growth that began at day 7 following treatment that continued to the end of the study. The addition of low dose XRT to M002 treatments had no significant effect upon tumor growth.

The second set of *in vivo* experiments examined the effects of M002 upon G401 MRKT xenografts. G401 cells [2.5×10^6^ in Matrigel™ (BD Biosciences)], were injected subcutaneously into the right flank of female athymic nude mice. Once flank tumors reached a volume of 250 mm^3^, the tumors were injected with either control vehicle (PBS+glycerol, 50 µL, n = 5) or M002 (1×10^7^ PFU/50 µL, n = 10). Again, to determine if irradiation improved the tumoricidal effects of M002, the control animals and half of the M002 treated animals received a low dose of external beam irradiation (3 Gy, XRT) to the tumor following injection with vehicle or M002. The vehicle only control was omitted from this study to reduce the number of animals utilized since previous work in our laboratory showed no effect of low dose irradiation (3Gy) upon G401 flank xenografts (Figure **S2**). When flank tumors reached the parameters dictated by IACUC protocol, or at 86 days when the experiment was terminated, the animals were euthanized and the tumors harvested. In the G401 xenografts, there was a statistically significant increase in survival in animals treated with M002 or M002+ XRT compared to control animals treated with vehicle+XRT (Figure 6. **A**). In addition, change in tumor volumes were decreased in animals treated with M002 (Figure 6. **B**) as early as one week post-treatment and continued for the remainder of the study. There was no survival advantage provided by the addition of low dose XRT to the virotherapy (69.4±13.1 vs. 56.4±14.2 days, M002 vs. M002+ XRT, p = 0.5). In addition, there was no significant difference in change in tumor volume between tumors treated with M002 or M002+ XRT (3.4±2.2 mm^3^ vs. 5.8±0.6 mm^3^, M002 vs. M002+ XRT, p = 0.2).

**Figure 7:**
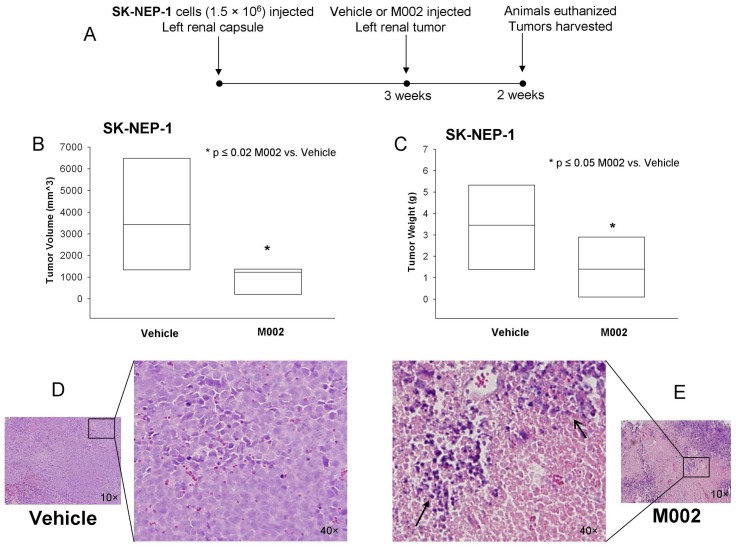
M002 treatment of renal Ewing sarcoma xenografts. **A** SK-NEP-1 cells, (1.5×10^6^ cells) were injected into the subcapsular space of the left kidney in 6 week old female athymic nude mice. After three weeks, the renal tumors were directly injected with either control vehicle (PBS +10% glycerol/50 µL, n = 6) or M002 oncolytic herpes simplex virus (1×10^7^ PFU/50 µL, n = 7). Two weeks following treatment, the animals were euthanized and the kidney tumors were harvested, measured with a caliper and weighed. **B** Tumor volumes in the animals treated with M002 were half those of animals treated with vehicle only (*bar = mean*). **C** Tumor weights following M002 treatment were also nearly half of those of vehicle treated tumors (*bar = mean*). **D** Formalin-fixed, paraffin embedded samples of vehicle treated SK-NEP-1 tumor specimens were stained for hematoxylin and eosin. Examination of these kidney tumor specimens revealed native tumor architecture consisting of sheets of small round blue cells. Representative photomicrographs at 10× with enlarged area of detail at 40×. **E** Formalin-fixed, paraffin embedded samples of M002 treated SK-NEP-1 tumor specimens were stained for hematoxylin and eosin. These specimens showed tumor necrosis (*open arrow*) and hemorrhage (*pink area*), and inflammatory cell infiltrates (*closed arrow*). Representative photomicrographs at 10× with enlarged area of detail at 40×.

**Figure 8:**
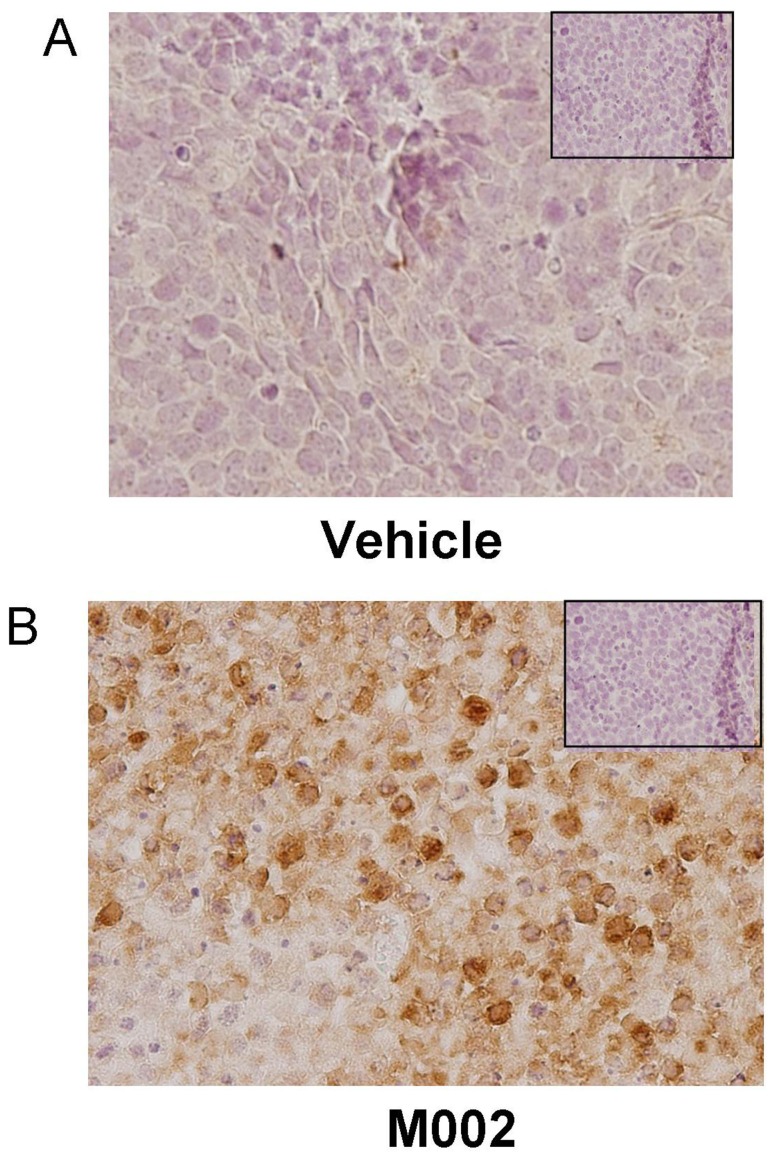
Immunohistochemical staining for HSV in SK-NEP-1 tumor xenografts. Formalin-fixed, paraffin embedded samples of SK-NEP-1 tumor xenografts (those presented in the data in Figure 5) were stained for HSV using immunohistochemistry, and representative photomicrographs presented. **A** No HSV was detected by immunohistochemical staining in SK-NEP-1 tumors treated with vehicle alone. Negative controls (rabbit IgG) were included with each run (*small box insert*). **B** HSV immunohistochemical staining revealed HSV present in the M002 treated SK-NEP-1 xenografts (*brown stain*). There was no viral staining seen in the negative controls (rabbit IgG) (*small box insert*).

Finally, we tested a murine model of renal Ewing sarcoma using the SK-NEP-1 cell line in nude mice (Figure 7.). SK-NEP-1 cells (1.5×10^6^) were injected into the subcapsular space of the left kidney, and after three weeks, the renal tumors were directly injected with either control vehicle (PBS +10% glycerol/50 µL, n = 6) or M002 (1×10^7^ PFU/50 µL, n = 7). Two weeks following treatment, the animals were euthanized and the kidney tumors were harvested, weighed, and measured with a caliper (Figure 7. **A**). Tumor volumes in the animals treated with M002 were half those of animals treated with vehicle only (1053.8±244.8 mm^3^ vs. 3623.4±1083.6 mm^3^, M002 vs. vehicle, p = 0.015) (Figure 7. **B**, *bar = mean*). Tumor weights following M002 treatment were also nearly half of those of vehicle treated tumors (1.51±0.45 g vs. 3.32±0.89 g, M002 vs. vehicle, p = 0.04) (Figure 7. **C**, *bar = mean*). Since kidney volume and weight may differ depending upon size of animal, animal weights were compared at the time of euthanasia between vehicle and M002 treated groups and there was no significant difference (25.0±0.9 g vs. 24.0±0.5 g, vehicle vs. M002, p = 0.4), therefore the differences in tumor volumes and weights were not related to decreased animal size secondary to oHSV treatment. Histological examination of the kidney tumor specimens (Figure 7. **D**, **E**) showed an increase in tumor necrosis (Figure 7. **E**, *open arrow*) and hemorrhage (Figure 7. **E**, *pink area*), inflammatory cell infiltrate (Figure 7. **E**, *closed arrow*) in the xenografts treated with M002 (Figure 7. **E**) and scattered tumor cells in comparison to those treated with vehicle alone (Figure 7. **D**). Xenografts that were treated only with vehicle retained their native tumor architecture consisting of sheets of small round blue cells (Figure 7. **D**). Formalin-fixed, paraffin embedded samples of SK-NEP-1 tumors were stained for HSV and Ki67 using immunohistochemistry. Two weeks after treatment, oHSV was still detected in the M002 treated tumor samples suggesting the virus continued to replicate (Figure 8. **B**, *brown staining*). Viral staining was not seen in the vehicle treated tumors (Figure 8. **A**) or in the negative controls (rabbit IgG, Figure 8. **A**, **B**, *inserts upper corner*). Cell proliferation as measure by Ki67 immunostaining tended to have less positive cells in xenografts treated with M002, but there was not a significant difference in the mean percentages between vehicle versus M002 treated xenografts (Figure **S3**, **S4**).

## Discussion

Despite advances in the treatment of pediatric cancers, long term survival for children with many solid organ tumors such as hepatoblastoma and rare renal tumors has not improved significantly and will clearly require novel therapies to alter this trend. Among the alternative treatments available are viral therapies. Investigators have utilized viruses both for their oncolytic properties as well as delivery systems to both liver and kidney tumor cells. Jana et al used a modified Sendai virus to successfully deliver nanoparticles to hepatocellular carcinoma cells demonstrating that this virus could serve as a drug delivery vehicle [30]. Measles virus engineered to produce carcinoembryonic antigen has been used to infect HuH6 cells. This engineered measles virus, acting as a vehicle for drug delivery, produced 60% cell death at MOI of 1 PFU/cell [31]. Chung showed that an oncolytic HSV decreased the growth of human hepatocellular xenografts [32]. Also, in a nude mouse model, Fu and colleagues utilized intratumoral injection of an HSV-2 virus engineered to induce cell fusion and showed its tumoricidal activity in renal cell carcinoma xenografts [14]. Finally, investigators have demonstrated that oHSVs will infect and have oncolytic effects in some sarcoma cell lines and xenografts [33], [34], [35].

An important aspect of the current study was the investigation of CD111 (nectin-1) in human hepatoblastoma and renal tumor cell lines and human tumor tissues. CD111 was detected by FACS in the hepatoblastoma cell line (HuH6) and in both of the renal tumor cell lines (G401 and SK-NEP-1). There appeared to be a correlation (Figure 3. **C**) between the percentage of cells that were CD111 positive (Figure 1. **B**) and resultant cell killing (Figure 3. **B**); the cell line with the highest CD111 expression (SK-NEP-1) required the lowest dose to kill 50% of the cells, whereas the cell line with the lowest CD111 (G401) required the highest dose. Previously, colleagues in our group demonstrated that CD111 was a critical entry molecule for oHSV in tumor cell lines, but found that not all tumors of the same histological type had sufficient levels of CD111 positive cells for oHSV to produce a relevant anti-tumor effect. They noted that for gliomas, levels of CD111 expression below 20% were associated with less of an oncolytic effect upon tumor cells resulting in decreased tumor cell killing compared to those with greater CD111 expression [26]. In the current study, however, despite lower levels of CD111 positivity in the G401 cell line, M002 successfully infected cells (Figure 2. **A**, **B**), decreased cell survival (Figure 3. **B**) and remained efficacious at decreasing tumor growth *in vivo* (Figure 6. **B**). These findings may be explained in a couple of ways. One explanation rests with the fact that infectivity and viral replication do not necessarily correlate with cell killing. Viral-induced cell killing is dependent upon numerous factors present in the hosts’ genome that contribute to the function of the virus, such as heat shock protein 27 (Hsp27) [36], STAT1 [18] and p38 [28]. Another explanation may be that the virus utilized an additional or different means for entry into the cells such as CD270, syndecan-2 or CD112. It has been reported in the literature that HSV-1 may use nectin-2 (CD112) for cell entry [37]. We examined the expression of CD112 in the cell lines utilized and this receptor was highly expresses in all three (Figure **S5**).

Another important feature of the current study was the examination of human specimens for the viral entry receptor CD111. In the human tumor specimens, the majority of both the hepatic and renal specimens investigated stained positive for CD111. To our knowledge, until this study, there have been no reports in the literature addressing the presence of nectin-1 in these tumor types. The expression of nectin-1 in these human specimens provided a basis for proceeding with the cytotoxicity studies and suggests that the humanized version of the M002 virus, M032, may be a beneficial therapeutic option for children with these difficult to treat solid tumors.

The number of cell lines available for study of these rare, but deadly, solid tumors is extremely limited. One of the cell lines that we chose to study for rare renal malignancies was the SK-NEP-1 cell line. This cell line was originally thought to be a Wilms tumor cell line, but has since been characterized as a renal Ewing sarcoma cell line [21]. This cell line was chosen for the current study since renal Ewing sarcomas, similar to MRKTs, are clinically much more difficult to treat and carry a significantly worse prognosis than the standard pediatric renal Wilms tumor that has five year survival rates exceeding 90% [38]. In the current study, we have seen that M002 had a significant oncolytic effect upon this cell line both *in vitro* and *in vivo*. A novel finding of these studies was the excellent response seen with M002 in SK-NEP-1 xenografts. The Seneca Valley Virus (NTX-010), a replication competent RNA virus, demonstrated only a low to intermediate response against the SK-NEP-1 cell line [39]. Treatment of other sarcoma types including rhabdomyosarcoma, osteosarcoma, Ewing sarcoma and malignant fibrous histiocytoma with oHSVs revealed that these sarcoma cell lines showed viral entry but varied in their sensitivity to viral oncolysis, with the Ewing sarcoma cells being the least susceptible [33], [34], [35].

Previously, investigators have reported that the addition of ionizing radiation to the administration of oHSVs produced a synergistic effect in xenograft models of lung cancer [40], mesothelioma [41], and cervical cancer [42] resulting in further decreased xenograft growth. Chung and colleagues studied this concept in hepatocellular carcinoma models [32]. They found that ionizing radiation (10 Gy) combined with R7020 recombinant oHSV resulted in a greater reduction in xenograft growth than either radiation or virus alone in one hepatocellular carcinoma cell line. The same results were not seen when they studied a second hepatocellular carcinoma cell line. We had similar findings in our study with HuH6 hepatoblastoma and G401 MRKT xenografts. The addition of ionizing radiation to oHSV therapy did not significantly affect xenograft growth compared to virus alone (Figure 5. **B**, Figure 6. **B**). We did utilize a lower dose of ionizing radiation than the Chung study, but our choice of radiation exposure (3 Gy) was based upon data that demonstrated a dose dependent increase in HSV replication following irradiation with 2 to 5 Gy, without any additional effects seen after 5 Gy [29]. In addition, it was felt that if an effect was seen with the addition of low dose ionizing irradiation, that perhaps dose reduction of both agents might be possible, having significant clinical implications.

It is important to note that there are no syngeneic mouse models available to study these tumor types. Therefore, immunodeficient murine xenograft models were chosen for the current studies in order to test the efficacy of M002 on human tumor cells *in vivo*. M002 was encoded with the gene for murine IL-12, best known as a T-lymphocyte and natural killer (NK) cell activator. Obviously, it was not possible to measure the immune contribution that the IL-12 had in tumor cell killing in our models since the mice were immunocompromised, but we believe that the addition of the IL-12 cytokine will produce a significant bystander effect in the clinical setting leading to tumor cells that were not infected by the virus to be destroyed by the immune system. Arming the oHSV with the immunomodulating cytokine, IL-12, offers a significant advantage to the virus alone since tumors are not uniform and tumor cells that have resistance to oHSV infection may be present in the tumor. There has been demonstration of this bystander effect in other studies utilizing immunocompetent mice [16], [18], [43]. There are other potential contributions afforded by the IL-12 in addition to the obvious immune modulatory effects in the setting of human therapeutics for some of these tumors. For example, alpha-fetoprotein (AFP) is produced in significant quantities by many hepatoblastomas. In hepatocellular carcinoma models, it was shown that AFP inhibited IL-12, leading to decreased cytolytic activity of NK cells, but reconstitution of IL-12 in the face of elevated AFP reversed these effects [44]. This study raises the hypothesis that in hepatoblastomas, the addition of IL-12 from the virus to the tumor bed may help to counteract some the effects of AFP upon the host’s immune system and lead to enhanced tumoricidal activity of the virus.

In both the hepatoblastoma (HuH6) and the malignant rhabdoid kidney tumor (G401) xenografts treated with M002, there was an initial shrinkage of the tumor followed by a small growth and eventual growth plateau. Importantly, the M002 treated tumors never reached the size of those xenografts that were treated simply with vehicle and XRT, and there were a few tumors that completely disappeared following M002 administration. There are a couple of potential explanations for these findings. First, the virus must come into contact with cells to infect them. Solid tumors have physical barriers such as blood vessels, connective tissue, areas of necrosis and hemorrhage that will prevent the virus from spreading to other cells. After the initial treatment, many of these physical barriers are enhanced as portions of the tumor undergo cell death, necrosis and subsequent scarring. The residual growth may be explained simply by a lack of the ability of the virus to physically reach these cells. Another potential explanation is again related to the host cells’ genome. Viral-induced cell killing is dependent upon numerous factors that contribute to the function of the virus such as STAT1 [18], p38 [28], and heat shock protein 27 (Hsp27) [36]. There is the possibility that one or more of these factors may be involved in the surviving tumor cells. These findings may have implications for the clinical treatment of solid tumors with virotherapy, suggesting that the virus may need to be administered repeatedly to the remaining tumor mass. Another option would be to give a low dose of irradiation to the remaining tumor after oHSV treatment. It has been shown in other tumor models that repeated low doses of irradiation without reinjection of virus resulted in continued viral replication with subsequent continued regression of the tumor [18], [28], [32]. Therefore, in the clinical setting, repeated injections of virus may be obviated by the addition of repeated low dose irradiation, making the use of oHSV treatment for disease located in sites that are not readily amenable to repeated injections, such as intrathoracic or intraabdominal tumors, more feasible.

We did examine cellular proliferation via Ki67 immunostaining as a potential measure of viral efficacy. There was not a significant difference in Ki67 staining seen in the SK-NEP-1 xenografts when comparing vehicle to M002 treated animals. These findings may be explained in a couple of different ways. First, the primary mechanism of action of oHSV was not on cell kinetics, but as a cytolytic agent. Therefore, proliferation may not be altered by oHSV treatment. Also, although the M002 treated xenografts were all significantly smaller than the treated xenografts, there likely remained viable tumor cells in the specimens that would naturally tend to proliferate. Finally, the method utilized to quantify the percentage of positive cells focuses upon the area in the specimen with the greatest immunoreactivity and does not account for intensity of immunostaining [22], thereby leading to areas of viable tumor in treated animals that may have a large percentage of the cells undergoing proliferation. An example of this point was demonstrated by Figure **S4** in the supporting information.

To summarize, we have shown for the first time that the oHSV, M002, was able to infect and replicate in human hepatoblastoma and aggressive pediatric renal tumor cell lines. To our knowledge, these tumor types have never before been tested against oHSV. Another novel aspect of this study was the demonstration that the entry mechanism for oHSV was present not only in these cell lines, but also in the majority of the human tumor specimens examined. Administration of M002 to murine xenograft models of hepatoblastoma, renal Ewing sarcoma, and malignant rhabdoid kidney tumors resulted in a significant increase in survival and a significant decrease in xenograft growth. These findings suggest that the humanized form of M002 oHSV may have therapeutic potential for children with these difficult to treat solid tumors.

## Supporting Information

Figure S1 **CD111 expression in glioma tumor cell lines.** Human glioma cell lines U251 and D54 were cultured under standard conditions using DMEM/F12 media supplemented with 10% FBS (HyClone) and 2.6 mM L-glutamine (Thermo Fisher Scientific). Cells were harvested using either trypsin or a cell scraper and stained for CD111. CD111 expression was quantified with FACS. The method of cell harvest did not significantly affect the expression of CD111.
(TIF)

Figure S2 **Irradiation treatment of malignant rhabdoid kidney tumor xenografts.** G401 human MRKT cells (2.5×10^6^ cells) in Matrigel™ were injected into the right flank of female athymic nude mice. Once tumors reached 250 mm^3^, animals received a sham treatment [anesthetized and placed in irradiator n = 5)] or low dose, 3 Gy, irradiation to the tumor (n = 5). Tumor volumes were measured twice weekly [(width)^2^×length]/2. Data reported as mean fold change in tumor volume ± standard error. Treatment with low dose XRT had no significant effect upon tumor growth.
(TIF)

Figure S3 **Immunohistochemical staining for Ki67 in SK-NEP-1 tumor xenografts.** Formalin-fixed, paraffin embedded samples of SK-NEP-1 tumor xenografts (those presented in the data in Figure 5) were stained for Ki67 as a measure of cellular proliferation. Slides were examined and percentage of positive cells quantified [22]. Although there tended to be less Ki67 staining in the M002 treated tumors, there was no significant difference in the mean percentage of positive cells between the vehicle and M002 treated xenografts (*bar = mean*).
(TIF)

Figure S4 **Immunohistochemical staining for Ki67 in SK-NEP-1 tumor xenografts.** Formalin-fixed, paraffin embedded samples of SK-NEP-1 tumor xenografts (those presented in the data in Figure 5) were stained for Ki67 using immunohistochemistry, and representative photomicrographs (40×) presented. There was cellular proliferation detected in both vehicle (*left panel*) and M002 (*right panel*) treated xenografts (*dark brown staining, arrows*). Negative controls (rabbit IgG) (*small insert corner left panel*) and normal kidney (*small insert corner right panel*) reacted appropriately.
(TIF)

Figure S5 **CD112 (nectin-2) expression in hepatoblastoma and rare pediatric renal tumor cell lines.** Human cell lines HuH6, G401, and SK-NEP-1 were stained with fluorescence antibody for CD112 and evaluated by FACS. CD112 staining was detected in all three of these cell lines.
(TIF)
