# Supplementary figures and images for: Preclinical Evaluation of Engineered Oncolytic Herpes Simplex Virus for the Treatment of Pediatric Solid Tumors
Source: PLoS One. 2014 Jan 30;9(1):e86843. doi: 10.1371/journal.pone.0086843 (PMC3907427; doi:10.1371/journal.pone.0086843)

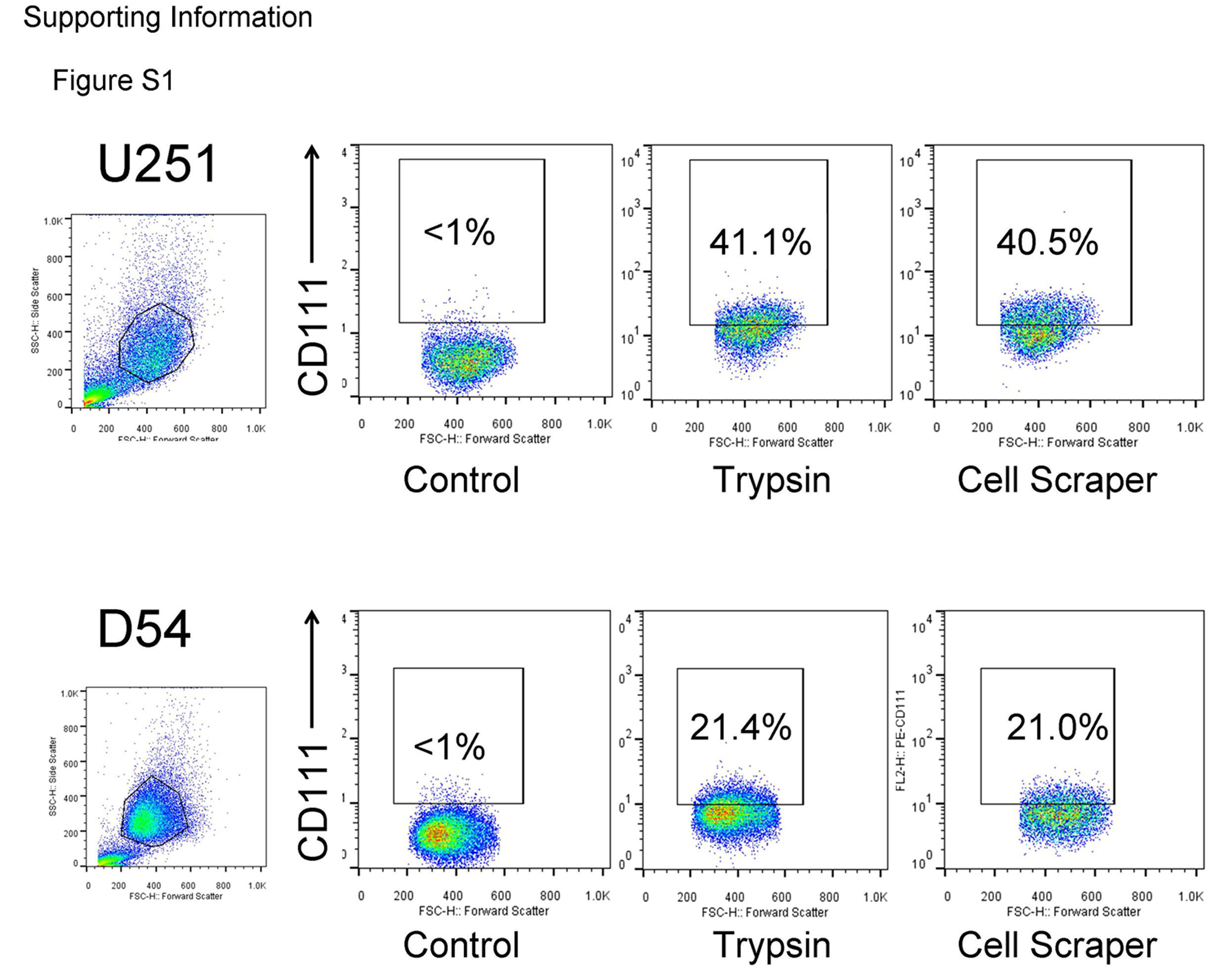

Supplement: Figure S1 — CD111 expression in glioma tumor cell lines. Human glioma cell lines U251 and D54 were cultured under standard conditions using DMEM/F12 media supplemented with 10% FBS (HyClone) and 2.6 mM L-glutamine (Thermo Fisher Scientific). Cells were harvested using either trypsin or a cell scraper and stained for CD111. CD111 expression was quantified with FACS. The method of cell harvest did not significantly affect the expression of CD111. (TIF) [file pone.0086843.s001.tif]

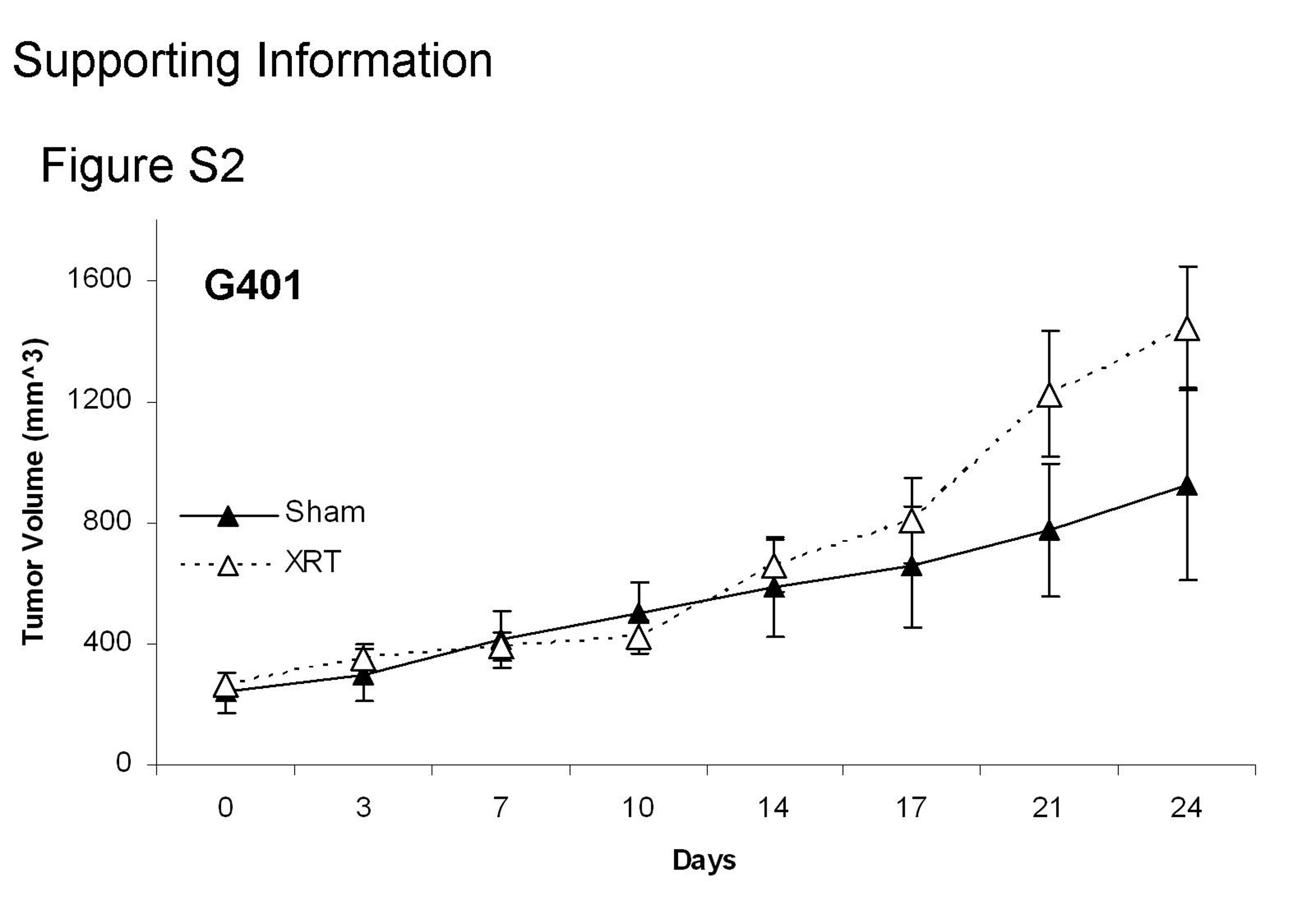

Supplement: Figure S2 — Irradiation treatment of malignant rhabdoid kidney tumor xenografts. G401 human MRKT cells (2.5×10^6^ cells) in Matrigel™ were injected into the right flank of female athymic nude mice. Once tumors reached 250 mm3, animals received a sham treatment [anesthetized and placed in irradiator n = 5)] or low dose, 3 Gy, irradiation to the tumor (n = 5). Tumor volumes were measured twice weekly [(width)2×length]/2. Data reported as mean fold change in tumor volume ± standard error. Treatment with low dose XRT had no significant effect upon tumor growth. (TIF) [file pone.0086843.s002.tif]

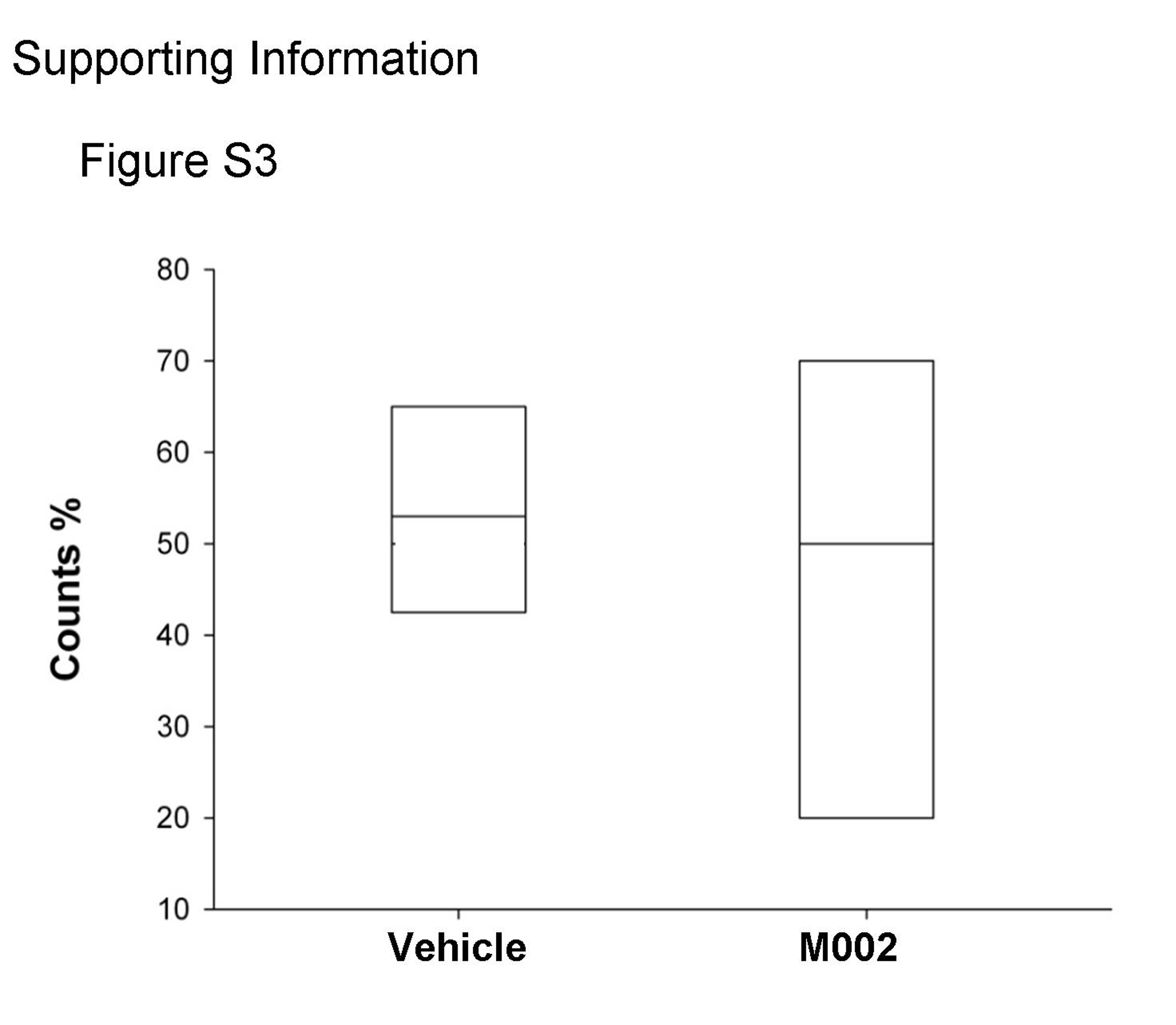

Supplement: Figure S3 — Immunohistochemical staining for Ki67 in SK-NEP-1 tumor xenografts. Formalin-fixed, paraffin embedded samples of SK-NEP-1 tumor xenografts (those presented in the data in Figure 5) were stained for Ki67 as a measure of cellular proliferation. Slides were examined and percentage of positive cells quantified [22]. Although there tended to be less Ki67 staining in the M002 treated tumors, there was no significant difference in the mean percentage of positive cells between the vehicle and M002 treated xenografts (bar = mean). (TIF) [file pone.0086843.s003.tif]

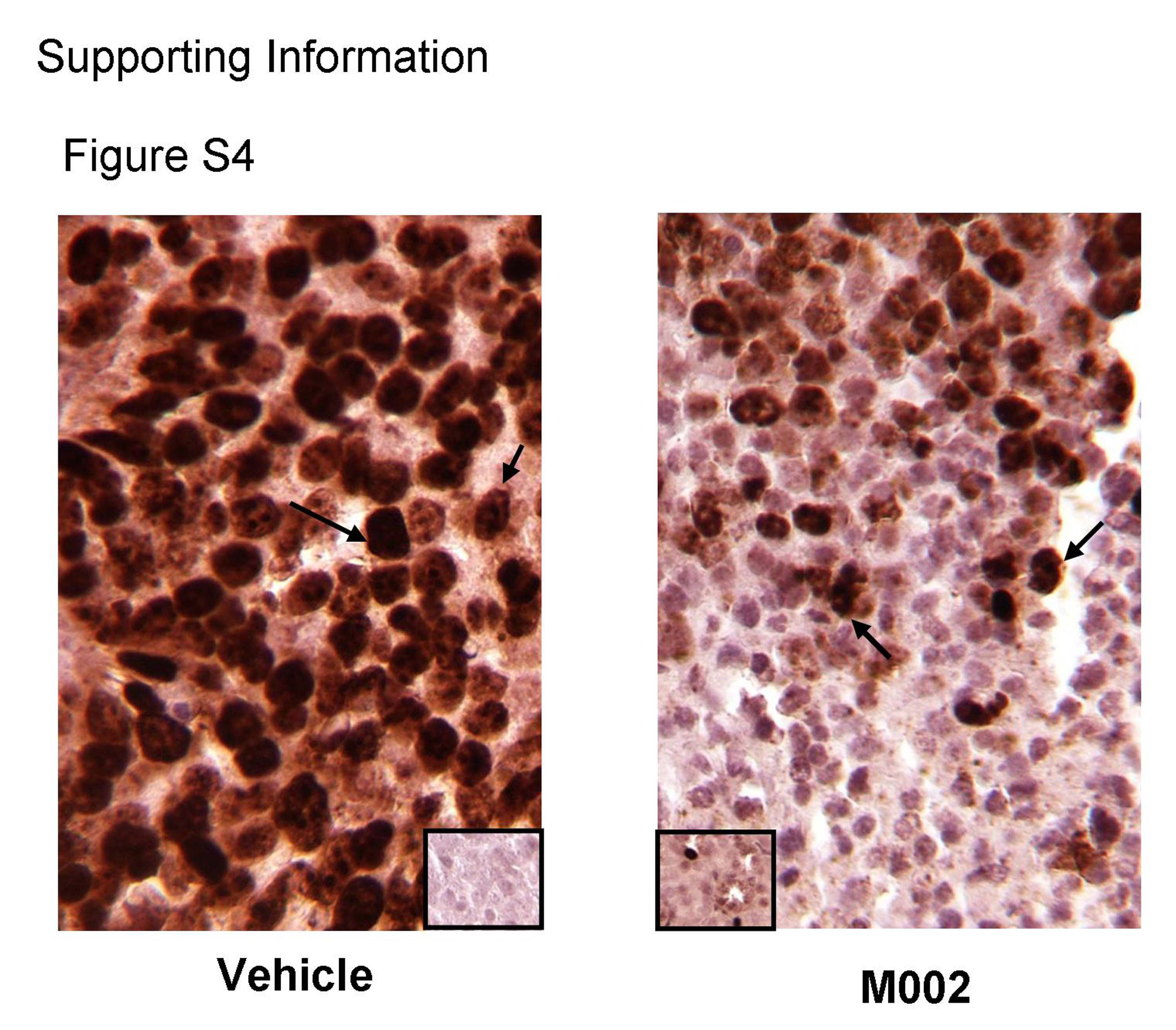

Supplement: Figure S4 — Immunohistochemical staining for Ki67 in SK-NEP-1 tumor xenografts. Formalin-fixed, paraffin embedded samples of SK-NEP-1 tumor xenografts (those presented in the data in Figure 5) were stained for Ki67 using immunohistochemistry, and representative photomicrographs (40×) presented. There was cellular proliferation detected in both vehicle (left panel) and M002 (right panel) treated xenografts (dark brown staining, arrows). Negative controls (rabbit IgG) (small insert corner left panel) and normal kidney (small insert corner right panel) reacted appropriately. (TIF) [file pone.0086843.s004.tif]

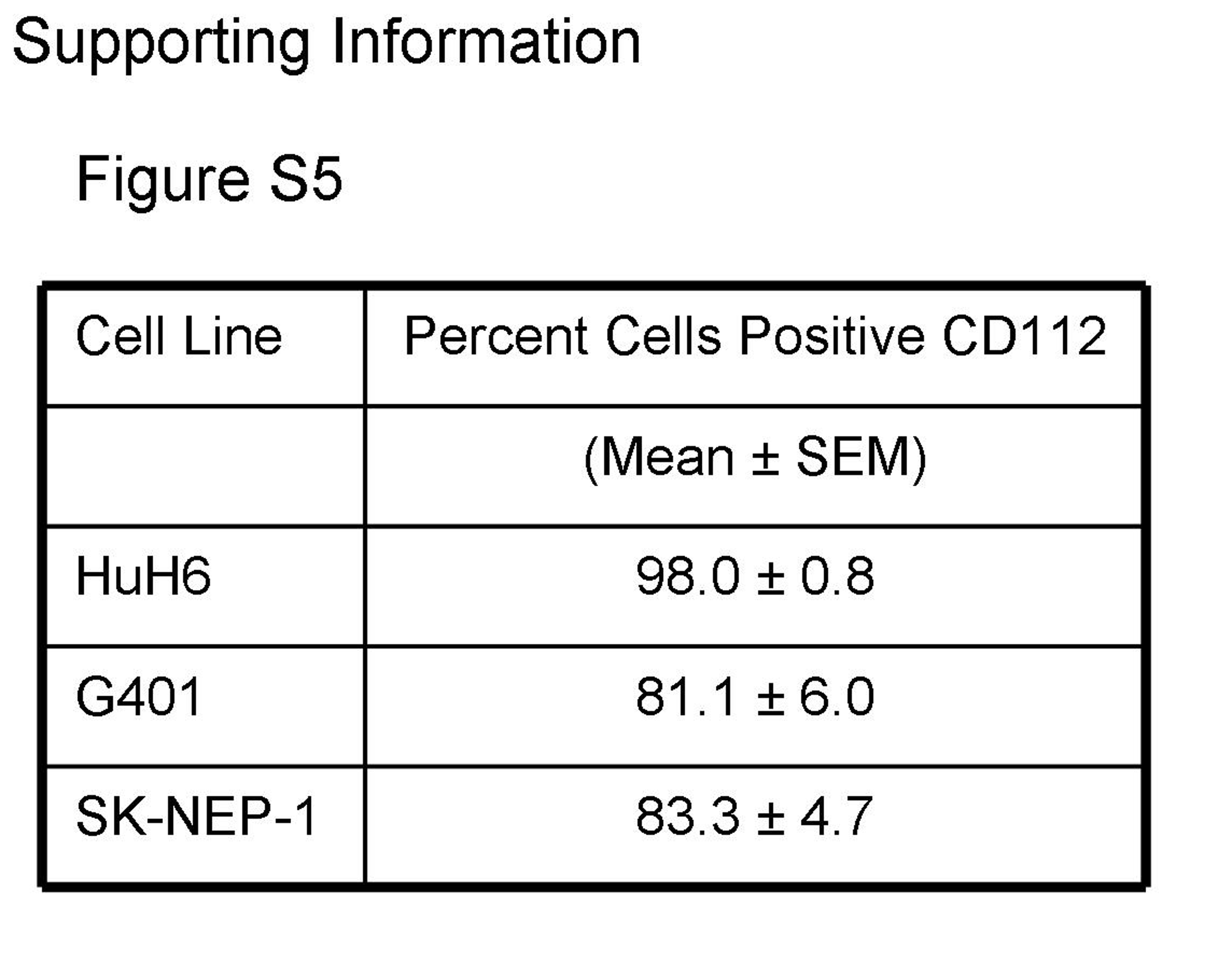

Supplement: Figure S5 — CD112 (nectin-2) expression in hepatoblastoma and rare pediatric renal tumor cell lines. Human cell lines HuH6, G401, and SK-NEP-1 were stained with fluorescence antibody for CD112 and evaluated by FACS. CD112 staining was detected in all three of these cell lines. (TIF) [file pone.0086843.s005.tif]
